# Implementation of a Fuzzy Inference System to Enhance the Measurement Range of Multilayer Interferometric Sensors

**DOI:** 10.3390/s22176331

**Published:** 2022-08-23

**Authors:** Everardo Vargas-Rodriguez, Ana Dinora Guzman-Chavez, Rafael Guzman-Cabrera, Anderson Smith Florez-Fuentes

**Affiliations:** 1 Departamento de Estudios Multidisciplinarios, Universidad de Guanajuato, Yuriria 38940, Guanajuato, Mexico; 2 Departamento de Ingeniería Eléctrica, Universidad de Guanajuato, Salamanca 36885, Guanajuato, Mexico

**Keywords:** optical sensors, interferometry, optical filters, temperature sensors, Fabry–Perot interferometer, fuzzy logic

## Abstract

This work presents a novel methodology to implement a fuzzy inference system (FIS) to overcome the measurement ambiguity that is typically observed in interferometric sensors. This ambiguity occurs when the measurand is determined by tracing the wavelength position of a peak or dip of a spectral fringe. Consequently, the sensor measurement range is typically limited to the equivalent of 1 free spectral range (FSR). Here, it is demonstrated that by using the proposed methodology, the measurement range of this type of sensor can be widened several times by overcoming the ambiguity over some FSR periods. Furthermore, in order to support the viability of the methodology, it was applied to a couple of temperature interferometric sensors. Finally, experimental results demonstrated that it was possible to quintuple the measurement range of one of the tested sensors with a mean absolute error of MAE = 0.0045 °C, while for the second sensor, the measurement range was doubled with an MAE = 0.0073 °C.

## 1. Introduction

Sensors based on interferometric filter heads have been widely used for a long time, and these can be used in a broad range of applications. In the literature, sensors can be found for measuring physical variables such as pressure [1,2,3], temperature [4,5,6], refractive index [7,8,9,10], curvature [11,12,13] and displacement [14]. Moreover, in many of these sensors, the characteristics of their reflection or transmission intensity distribution spectra are affected by changes in the measurand. Therefore, it is quite popular to monitor one spectral feature to establish relationships between it and the measurand. For instance, in some sensors, their corresponding spectra present changes in the amplitude of fringes as the measurand is varied. For this kind of sensor, the spectral feature that can be monitored is the peak or the dip amplitude of one fringe. Some examples of this case are the refractive index sensors proposed by [8,9,10], where authors were able to define the RI as a function of the dip of one fringe. Moreover, there are other sensors in which the position of spectral fringes is shifted as the measurand is varied. For these kinds of sensors, it is quite popular to take the wavelength position of a fringe peak or dip as the spectral feature of reference to determine the measurand [1,4,15]. For instance, Novais et al. in 2018 presented a sensing arrangement for detecting water–glycerin mixtures. Their sensor was based on a Fabry–Perot interferometer (FPI) which was formed by an air micro-bubble fabricated at the tip of a single mode fiber (SMF). Moreover, the reflection spectrum of the FPI exhibited a spectral shift depending on the overall effective refractive index of the mixture. For the mentioned sensor, the authors were able to establish linear relationships between the wavelength positions of some fringe peaks and the water mass fraction in glycerin [15]. Another interesting sensor capable of measuring pressure or temperature has been proposed by Zhang et al. in 2021 [1], in which both parameters are described as linear functions of the wavelength positions of some dips. In the reported spectra of that work, it can be clearly observed that the wavelength position of one dip is shifted to the right as the temperature increases, but when it reaches a certain level, the dip wavelength practically returns to its initial position. This is a normal effect observed in FPI sensors, and it is due to the fact that a particular fringe has been shifted more than 1 free spectral range (FSR), reaching the spectral region of a neighbor fringe. Its effect raises an ambiguity problem, since the wavelength position of a fringe peak or dip can be the same for different temperature levels. Consequently, when the single wavelength position is considered as the spectral feature, the measurement range is usually limited by 1 FSR period. In another example, a pressure sensor based on a double FPI arrangement at the tip of an SMF has been presented by Zhu et al. in 2021 [2], in which the overall reflection spectrum is formed by the superposition of two interference spectra. Moreover, the authors computed the envelope of the overall spectrum and traced the wavelength position of one dip. Afterwards, the pressure was described as a linear function of the wavelength position of the envelope dip. In another work, a gas pressure sensor, based on an interferometric head at the tip of an SMF, was presented by Liu et al. in 2021 [3]. In that sensor, the overall reflection spectrum is formed by the superposition of different spectra, and therefore, the authors applied low and high-pass filters in order to separate its components. In this way, they obtained two spectra, which were called the resonant and the interference spectra. Afterwards, the gas pressure was a function of the refractive index of the gas and the temperature. Hence, the wavelength position of one fringe peak of the resonant spectra and one from the interference spectra were traced. Later, they used these wavelength shifting with a two-parameter sensitivity matrix to determined RI and temperature simultaneously. Moreover, authors described that the measurement range will be limited to 1 FSR by the ambiguity. Therefore, they suggested the use of an additional demodulating stage based on fiber Bragg grating, which will be keeping working continuously (24 h), in order to be able to overcome this ambiguity. In this way, they were able to perform correct measurements outside the 1 FSR limit, and consequently, in practice, the measurement range was widened.

Here, it is important to point out that different techniques of artificial intelligence have been become quite popular due to their potential to solve complex and nonlinear models. Particularly, in the literature, there can be found several works reporting the use of machine learning techniques to enhance particular characteristics of photonic systems [16] and optical sensors [17]. As an example, Manuel et al. in 2022 used a support vector machine to overcome the intensity ambiguity observed when the refractive index is determined based on the Fresnel reflection occurring at the tip of a fiber [18]. In a further example, recently, researchers proposed a method based on an ensemble of artificial neural networks (ANN) to widen the measurement range of a temperature sensor. In that work, they required ensembles with more than 40 ANNs members to be able to double the measurement range of the sensor with a mean absolute error (MAE) = 0.17 °C [19]. Moreover, fuzzy inference systems (FIS) are another powerful option to solve complex problems. In the literature, there can be found applications of FIS in practically all fields. For example, they have reported FIS for applications such as the prediction of powerful earthquake parameters [20], the assessment of indoor air quality in operation rooms [21], and the prediction of cloacal temperature of broiler chickens [22]. Optical-related applications have been reported as well by Wang et al. in 2021, who implemented a hybrid method based on Taguchi and fuzzy logic to effectively optimize the characteristics of a composite optical receiver [23]. Additionally, a similar Taguchi–fuzzy logic hybrid approach has been proposed to optimize the mechanical properties for cutting polyester fiber with ultraviolet (UV) light [24].

In this work, we present a novel methodology to implement an FIS to overcome the ambiguity that will be observed when spectral fringes are shifted more than 1 FSR. Consequently, by using this methodology, the measurement range of interferometric sensors can be widened several times. Moreover, we presented the procedures followed to form all required membership functions to implement the FIS. Furthermore, in order to support the viability of the proposed methodology, we applied it to two temperature interferometric sensors. These sensors were numerically simulated and experimentally implemented, and from their corresponding spectra, different spectral features were extracted. Additionally, it is shown that the number of spectral features that will be considered as inputs of the FIS will depend on the interferometric filter design and on how many times the measurement range will be widened. Based on experimental results, it is shown that by using the proposed methodology for one of the sensors, the temperature was correctly determined over 2 FSR periods when only two spectral features were considered. For this case, the measurement range was doubled, and the estimated temperatures presented a mean absolute error (MAE) = 7.4 × 10${}^{-3}$ °C and a mean squared error (MSE) = 8.1 × 10${}^{-8}$ (${}^{{}^{\circ}}$C)${}^{2}$. Additionally, for the second sensor, its measurement range was quintupled, since it was possible to overcome the ambiguity within 5 FSR periods. For this case, we considered five spectral features as inputs of the FIS, and the estimated temperatures presented an MAE = 4.5 × 10 ${}^{-3}$ °C and an MSE = 3.0 × 10${}^{-5}$ (${}^{{}^{\circ}}$C)${}^{2}$. As can be expected, the results obtained when the methodology was applied over the synthetic datasets were also successful and presented better MAE and MSE figures. Finally, it should be pointed out that this methodology has the advantage that can it be applied to enhance the measurement range of similar interferometric sensors without the need to change their physical setups.

## 2. Optical Sensing Setup

**Figure 1:**
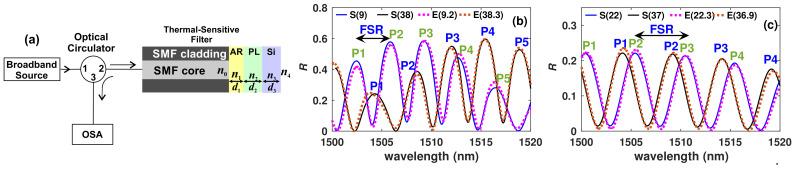
(**a**) Sensing arrangement and the interferometric filter head; simulated (S) and measured (E) reflected intensity distribution spectra of (**b**) sensor 1 and (**c**) sensor 2 at different temperatures.

The implemented optical sensor setup is shown in (Figure 1a). Here, the light reflected by the interferometric optical filter is recorded by an optical spectrum analyzer (OSA). Moreover, as it can be appreciated (Figure 1a), the filter design is based on a three-layer stack deposited at the tip of a single mode fiber (SMF). From these, the thickness and the refractive index of the polymer (PL) and the silicon (Si) layers are affected by temperature. Consequently, the overall reflection spectrum of the filter is temperature dependent. Simulated and experimentally measured reflected intensity distribution spectra of the sensor considering different interferometric filters and temperatures are presented in Figure 1b,c. Here, the design of both filters is the same, but the thickness of the Si and PL layers are different. The mathematical model and the fabrication procedure of these filters have been discussed in detail previously [25].

The fabrication procedure of the interferometric optical filter consisted mainly of the following steps: (a) a ferrule sleeve that matches a single mode pigtailed ferrule (Thorlabs SMPF0215-FC) was in-house fabricated; (b) a segment of a double-sided polished silicon wafer was fixed at the bottom of the ferrule sleeve, and it was kept in a vertical position by means of a ferrule clamp; (c) the single-mode pigtailed ferrule with a 0° face and an antireflection coating was introduced by the top side of the ferrule sleeve; (d) the experimental setup shown in Figure 1a was implemented in order to monitor the reflection spectrum; (e) the position of the pigtailed ferrule within the sleeve was controlled with a three-axis translation stage (Thorlabs RB13M/M); (f) by displacing the pigtailed ferrule, a cavity between its face and the silicon wafer is formed; (g) the length of the cavity was estimated by measuring the FSR of the fringes of the reflection spectrum; (g) once the desired cavity length is set, the liquid cyanoacrylate polymer is dropped by the top side of the ferrule sleeve in such a way that it flows to the bottom filling the cavity; (h) the polymer is cured in a few minutes at ambient temperature. Following this process, the layer thicknesses of the fabricated filters were, for filter 1: $d_{1}=0.3229$, $d_{2}=37.02$ and $d_{3}=$ 84.95 μm, while for filter 2: $d_{1}=0.3229$, $d_{2}=149.80$ and $d_{3}=$ 6.15 μm. Moreover, the thermo-optic and thermo-expansion coefficients of layers 2 and 3 were $\rho_{2}=$0.4 × 10${}^{-4}$ K${}^{-1}$, $\rho_{3}=$ 1.88 × 10${}^{-4}$ K${}^{-1}$, $\gamma_{2}=$ 198 μm/(mK) and $\gamma_{3}=$ 2.6 μm/(mK), respectively. Finally, the refractive indexes of the layer’s materials were $n_{0}=1.44$, $n_{1}\approx 1.2$, $n_{2}=1.45$, $n_{3}\approx 3.4$ and $n_{4}=1$ at 398 K.

**Figure 2:**
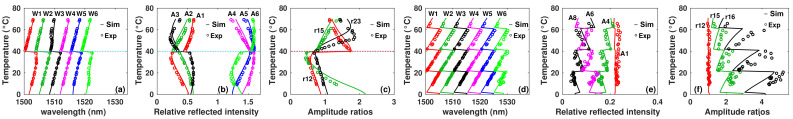
Graphical comparison between a few simulated and measured: (**a**,**d**) wavelength peak positions, (**b**,**e**) peaks amplitudes, (**c**,**f**) amplitude ratios of filters 1 and 2, respectively; in (**b**) A4–A6 are shifted right 1 unit for clarity purposes.

The reflection spectrum of these filters will be composed by the superposition of spectra which occur due to multiple reflections within the Fabry–Perot cavities. Moreover, as the temperature increases, spectral fringes will shift to the right, and consequently, for a given temperature level, these will reach the position of their neighbor fringes. For instance, in Figure 1c, it can be clearly seen that fringe P1 at 37 °C almost reaches the position that has the fringe P2 at 22 °C. In practical applications, these filters can be used as temperature sensors, since a relationship between the wavelength position of one fringe and the temperature can be established. However, the main disadvantage of these sensors will be their nominal measurement range, which will be limited to 1 FSR (Figure 1). In order to avoid this limitation, a novel methodology based on fuzzy logic is proposed, and it will be tested considering the simulated and experimentally measured spectra of these two sensors. Furthermore, Figure 2 presents a graphical comparison between some simulated and measured spectral features of the two optical filters.

## 3. Methodology for Predicting the Measurand Based on Fuzzy Logic

### 3.1. Fuzzy Inference Systems

In many real applications, the information that describes the corresponding processes is incomplete or it has uncertainties. These kinds of situations usually are observed in processes that involve humans due to our inherent cognitive processes [26]. The fuzzy sets, introduced by Zadeh (1965) [27], are a powerful alternative to represent and to manipulate these kinds of imprecise data. Additionally, fuzzy logic is a set of mathematical principles that provides a conceptual framework to deal with the problem of uncertainty and lexical imprecision [20,26].

Moreover, a fuzzy inference system (FIS) is an intelligent technique that usually is suitable for dealing with applications where uncertain or approximate reasoning is observed and for systems for which its mathematical model is difficult to derive [21,26]. In general terms, an FIS allows to map an input space into an output space by using fuzzy logic. The general structure of an FIS is formed by the following modules:

(a) Fuzzification interface: contains the fuzzification operators that will be used to transform the crisp values of the inputs variables into fuzzy sets.
(b) Fuzzy rule-base or knowledge base: a collection of fuzzy rules that can characterize the overall behavior of a fuzzy system when these are combined with sentences such as the connective also [26].
(c) Fuzzy inference machine: a mechanism that allows the modeling of the reasoning process based on the interpolation between the outputs of all fuzzy rules [28]. In this way, the fuzzy output corresponding to the fuzzified inputs can be determined. Moreover, the Mamdani, Tsukamoto, Sugeno and Larsen inference mechanisms are some of the most popular [26].
(d) Defuzzification interface: this module transforms the fuzzy output obtained by the inference machine into a crisp value, which usually is the overall output of an FIS [26].

**Figure 3:**
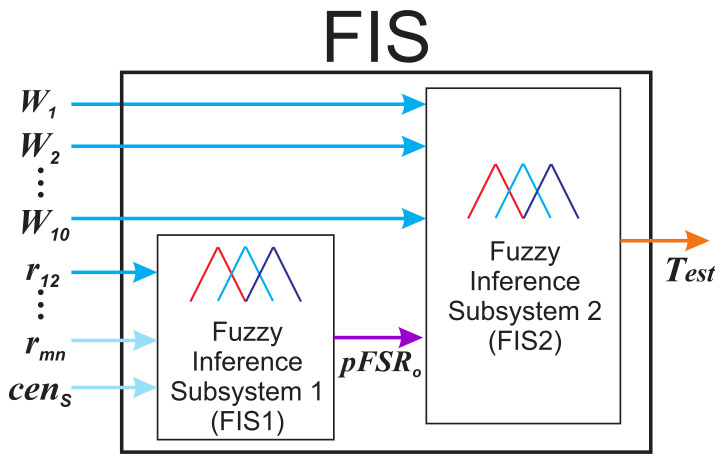
Block diagram of the proposed FIS.

Now, based on this conceptual frame, we can define that in our sensor, the measurand will be determined by implementing a fuzzy inference system (FIS). In our methodology, the FIS was composed by two fuzzy inference subsystems; this was useful to simplify the overall procedure. The first fuzzy inference subsystem (FIS1) is intended to determine the period of the FSR corresponding to a given combination of input variables, and it is based on a Mamdani inference mechanism. The second fuzzy inference subsystem (FIS2) will predict the final sensor output, which in our case will be temperature, based on the output of the FIS1 and the wavelength positions ($W_{x}$) of one or more fringe peaks. The FIS2 is based on the Tsukamoto mechanism. The main block diagram of the proposed methodology is presented in Figure 3.

**Figure 4:**
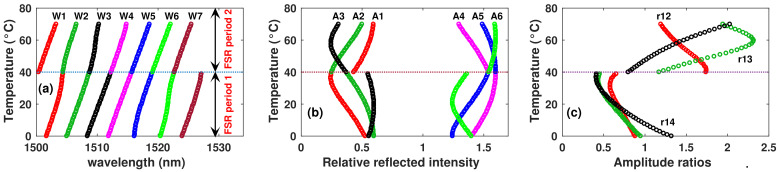
Simulated (**a**) peak wavelength positions, (**b**) peak amplitudes (**c**) amplitudes ratios as a function of temperature of sensor 1. In (**b**), A4–A6 were shifted 1 unit to the right for clarity purposes.

In order to explain the methodology we will take as a case of study the simulated dataset of observations for the filter 1. For this case, the wavelength positions ($W_{x}$) and the amplitude ($A_{x}$) of fringes $P_{1}$ to $P_{6}$ as a function of the temperature are presented in Figure 4a,b, respectively. Here, it can be observed that practically within the temperature range 0 ≤ *T* ≤ 70 °C, there are two periods of FSR. At the point where a change occurs in the FSR period, it can be observed that there is a kind of discontinuity in the trace of the wavelength positions (Figure 4a). Moreover, regarding the amplitude of fringes, not all have a clear and simple pattern, as in the case of wavelength positions. However, for this filter, it can be observed that the amplitude of peak $A_{1}$ > $A_{2}$ during the first period of FSR (pFSR), while $A_{1}$ < $A_{2}$ for the second pFSR. By taking advantage of this feature, the amplitude ratio $r_{12}$ = $A_{1}$/$A_{2}$ can be computed and considered as a spectral feature (Figure 4c). There are other amplitude ratios that satisfy the condition to be ≥1 during one pFSR and <1 within the other pFSR (i.e., $r_{13}$). However, it must be pointed out that not all the ratios satisfy this condition: for instance, $r_{14}$ (Figure 4c). Here, a spectral feature such as $r_{12}$ can be useful to determine the period of FSR, and therefore, it can be considered as good input of the FIS1. However, in some cases, it is not possible to find one variable that satisfies this condition, and therefore, a combination of more spectral features must be considered. In our methodology, once the correct period of the FSR (pFSRo) is known, the final output can be estimated by considering the wavelength position of one or more spectral fringe peaks. For clarity purposes, in the next sections, the procedure described will be followed to implement each one of the fuzzy inference subsystems (FIS1 and FIS2).

### 3.2. Fuzzy Inference Subsystem 1 (FIS1)

The FIS1 will interpret the values presented at input variables, and based on a set of fuzzy rules, it will estimate a value to the output variable. The inputs of FIS1 will be some spectral features that can help us determine the correct FSR period. In our case, these variables were some amplitude ratios and the centroid of the reflected spectrum. Moreover, this FIS1 will infer the correct period of the FSR ($pFSR_{o}$) that corresponds to the temperature of the filter based on the input values. In order to implement this FIS1, it is important to build some membership functions depending on the inputs and output variables. Moreover, it is important to know the inference mechanism that will be used. Therefore, in the next subsections, these issues will be explained.

#### 3.2.1. Membership Functions Used to Fuzzificate the Input Variables

In general, all membership functions used to fuzzificate the inputs of FIS1 were built by using the procedure described in Algorithm 1.
**Algorithm 1** Procedure for building the membership functions to fuzzificate the inputs variables of FIS1
  1. Initialize a counter of input variables (*n* = 1);  2. Define one spectral feature that will be considered as an input, it will be identified as $I_{n}$. For instance, $I_{1}=r_{12}\left(A_{1},A_{2}\right)$;  3. Set the number of regions (*N*) in which the universe of discourse of the input variable will be partitioned;  4. Locate within the observations dataset the maximum value of the input variable and set its upper limit $u_{n\max}$ = 1.05 ×$I_{n\max}$;  5. Declare the universe of discourse of the input variable, as $U_{n}=\left[0,u_{n\max}\right]$;  6. A set of *N* membership functions ($MFI_{n}^{1}$, …, $MFI_{n}^{N}$) are formed based on the following definitions:(1)$$\begin{array}{c} MFI_{n}^{1}\left(I_{n}\right)=\left(-\infty ,-\infty ,tc_{1}-\Delta I_{n},tc_{1}+\Delta I_{n}\right)\\ MFI_{n}^{2}\left(I_{n}\right)=\left(tc_{1}-\Delta I_{n},tc_{1}+\Delta I_{n},tc_{2}-\Delta I_{n},tc_{2}+\Delta I_{n}\right)\\ \vdots \\ MFI_{n}^{N-1}\left(I_{n}\right)=\left(tc_{N-1}-\Delta I_{n},tc_{N-1}+\Delta I_{n},tc_{N}-\Delta I_{n},tc_{N}+\Delta I_{n}\right)\\ MFI_{n}^{N}\left(I_{n}\right)=\left(tc_{N}-\Delta I_{n},tc_{N}+\Delta I_{n},+\infty ,+\infty ,\right) \end{array}$$   where $tc_{i}$ are the corners of the membership functions and can be evaluated as $tc_{i}=i$$I_{n\max}$/*N*;  7. Save the set of membership functions for this input variable.  8. If the first fuzzy logic stage considers another input variable, increment the counter n=n+1 and repeat steps 2 to 8.

#### 3.2.2. Membership Functions Related to the Output Variable of FIS1

The number of the period of FSR will be the single output of the FIS1, and it will be represented by the variable pFSR. Hence, it is needed to define its associated membership functions. Firstly, based on the experience that we have of the sensor behavior, it needed a membership function for each FSR period observed within a given measurement range. The procedure to build these membership functions is described in Algorithm 2.
**Algorithm 2** Procedure for building the membership functions related to the output variable of FIS1  1. Declare the overall measurement range, for example $T_{\min}$ ≤ *T* ≤ $T_{\max}$;  2. Declare the number of periods of FSR (*K*) that can be observed within the given measurement range;  3. Set the universe of discourse of the output variable (pFSR) as [0,K];  4. The number of partitions of the universe of discourse is defined as *K*;  5. The *K* membership functions will have trapezoidal form, with a width of 1, and can be described as:
(2)$$\begin{array}{c} MFO_{1}^{1}\left(pFSR\right)=\left(-\infty ,-\infty ,1-b_{1},1+b_{1}\right)\\ MFO_{1}^{2}\left(pFSR\right)=\left(1-b_{1},1+b_{1},2-b_{1},2+b_{1}\right)\\ \vdots \\ MFO_{1}^{K-1}\left(pFSR\right)=\left(K-2-b_{1},K-2+b_{1},K-1-b_{1},K-1+b_{1}\right)\\ MFO_{1}^{K}\left(pFSR\right)=\left(K-1-b_{1},K-1+b_{1},+\infty ,+\infty \right) \end{array}$$


**Figure 5:**
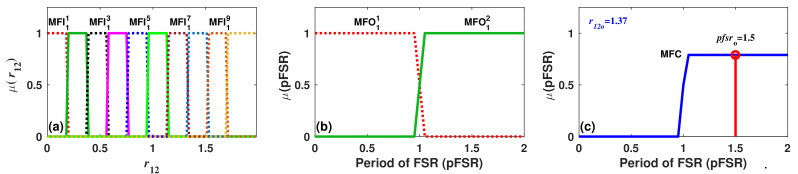
(**a**) Membership functions that will fuzzificate the input variable $r_{12}$ of filter 1; (**b**) Membership functions related to output variable (period of FSR) of FIS1; (**c**) Example of a membership function of the consequence for a particular value of $r_{12o}$ at its centroid.

As an example of the application of Algorithm 2, let us consider the case of filter 1. Here, by plotting the wavelength positions of the fringe peaks, it can be observed that all these traces present a discontinuity at 40 °C. This implies that two FSR periods occur over the measurement range 0 ≤ *T* ≤ 70 °C, and therefore, *K* = 2. The first FSR period occurs within the range 0 ≤ *T* ≤ 40 °C, while the second FSR period occurs when 40 < *T* ≤ 72 °C. Therefore, following Algorithm 2, the universe of discourse of the output variable can be set as $U_{pFSR}=\left[0,2\right]$, and it will be partitioned into K=2 regions. Finally, a set of K=2 trapezoidal membership functions, ($MFO_{1}^{1}$ and $MFO_{1}^{K}$), were formed by using definitions expressed in Equation (2) with a coefficient $b_{1}$ = 0.05 (Figure 5b).

#### 3.2.3. Fuzzy Rules, Inference Mechanism and Deffuzzification of FIS1

Based on the knowledge about the behaviour of the sensor, a set of *Q* IF–THEN fuzzy rules must be defined. Moreover, for the FIS1, these rules were evaluated by using a Mamdani implication operator. In this way, for each one of the *Q* fuzzy rules, a membership function of consequence is obtained. Once all membership functions of consequences ($MFC_{1}^{1}$, …, $MFC_{1}^{Q}$) have been evaluated, these are aggregated by using a max operator. This aggregation operation will generate an overall membership function for the consequence (MFC); this process can be expressed by using Equation (3). Finally, the MFC was defuzzified by means of the center-of-area or centroid [26] in order to obtain the deterministic value of the output ($pfsr_{o}$) of the FIS1.

(3) $$MFC\left(pFSR\right)=\max[MFC_{1}^{1}\left(pFSR\right),\ldots ,MFC_{1}^{Q}\left(pFSR\right)]$$

As an example of the application of these steps, let us continue with the case of filter 1. For this case, two rules (*Q* = 2) can be defined, straightforward relating the values of variable $r_{12}$ with the period of FSR. These rules are listed in Table 1. Moreover, following Mamdani’s implication operators, the rules can be computed as:

(4) $$\begin{array}{c} MFC_{1}^{1}\left(pFSR\right)=\min\left(\max\left(MFI_{1}^{1}\left(I_{1o}\right),MFI_{1}^{2}\left(I_{1o}\right),\ldots ,MFI_{1}^{5}\left(I_{1o}\right)\right),MFO_{1}^{1}\right),\\ MFC_{1}^{2}\left(pFSR\right)=\min\left(\max\left(MFI_{1}^{6}\left(I_{1o}\right),MFI_{7}^{2}\left(I_{1o}\right),\ldots ,MFI_{1}^{10}\left(I_{1o}\right)\right),MFO_{1}^{2}\right), \end{array}$$

where max and min are the maximum and minimum operators, respectively. In order to evaluate numerically these rules, let us consider a particular observation of our synthetic dataset, which contains the data listed in Table 2. Afterwards, all $MFC_{1}^{Q}$ functions are aggregated by using a max operator to generate the overall membership function for the consequence MFC (Figure 5c). Finally, it is defuzzified by evaluating the center-of-area or centroid; for this example, it is obtained that $pfsr_{o}$ = 1.5. This result indicates that the reflection spectrum has been shifted more than 1 FSR period when the temperature of the filter was increased by 59 °C.

### 3.3. Fuzzy Inference Subsystem 2 (FIS2)

**Figure 6:**
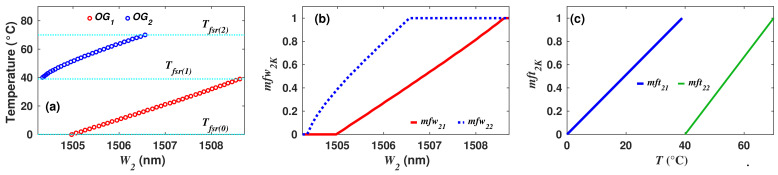
(**a**) Wavelength positions $W_{2}$ of peak $P_{2}$ as a function of temperature, and observations forming two groups corresponding to each one of the observed FSR periods; (**b**) membership functions $mfw_{2K}$ used to fuzzificate the wavelength position ($W_{2}$) of fringe’s peak $P_{2}$; (**c**) membership functions $wft_{xK}\left(T\right)$ to evaluate the fuzzy rules consequences.

In FIS2, the wavelength positions of *N* fringe peaks ($W_{x}$) of the interference spectrum and the pfsro (output of FIS1) are inputs variables. Furthermore, the FIS2 output will be the estimated temperature ($T_{est}$). Here, it is important to point out that within a segment corresponding to each period of FSR, the wavelength positions shifts in a monotonic way with respect to temperature (Figure 4a). Therefore, by taking advantage of these characteristics, it is possible to fuzzificate an input $W_{x}$ with a set of monotonic membership functions. In our case, these functions were built following Algorithm 3.
**Algorithm 3** Procedure for building the membership functions of FIS2  1. Initialize the counter *j* = 1;  2. Define the *x* fringe peak ($P_{x}$) for which its wavelength shifting ($W_{x}$) will be considered as an input of this FIS stage;  3. Locate all temperature intervals corresponding to each one of the *K* FSR periods observed within the overall temperature range. It can occur for more than one FSR period (for instance, see Figure 6a);  4. Split the original dataset observations into *K* groups ($OG_{1}$…$OG_{K}$). Observations corresponding to each FSR period must be in a particular OG group; it can be described as:
(5)$$\begin{array}{c} OG_{x1}=\left[T_{xfsr(0)}\leq T\leq T_{xfsr(1)},W_{xfsr(0)}\leq W_{x}\leq W_{xfsr(1)}\right]\\ \vdots \\ OG_{xK}=\left[T_{fsr(K-1)}\leq T\leq T_{xfsr(K)},W_{xfsr(k-1)}\leq W_{x}\leq W_{xfsr(K)}\right] \end{array}$$  5. Fit temperatures and wavelength positions of each one of the $OG_{xK}$ group observations. Here, a set of *K* two columns datasets [$T_{x1}$, $W_{x1}$], …, [$T_{xK}$, $W_{xK}$] will be formed corresponding to each one of the FSR periods;  6. Normalize the column $T_{xK}$ of each dataset in such a way that it has values between (0, 1], and label them as $\alpha_{x1}$, …, $\alpha_{xK}$.  7. Update the *K* datasets in the form [$T_{x1}$, $W_{x1}$, $\alpha_{x1}$], …, [$T_{xK}$, $W_{xK}$, $\alpha_{xK}$].  8. Build a set of *K* membership functions to evaluate the consequence of each rule in the form:
(6)$$\begin{array}{c} mft_{x1}=\left[T_{x1},\alpha_{x1}\right]\\ \vdots \\ mft_{xK}=\left[T_{xK},\alpha_{xK}\right] \end{array}$$  9. Within the *K* column arrays of $W_{xK}$, locate the minimum ($W_{x\min}$) and the maximum ($W_{x\max}$) wavelength positions of fringe peak $P_{x}$;  10. Define a new universe of discourse for the wavelength positions of fringe peak $P_{x}$ as $U_{Wx}$ = [$W_{x\min}-\Delta W_{x}$, $W_{x\max}+\Delta W_{x}$];  11. Build *K* new membership functions by using the expression:
(7)$$mfw_{xK}=\left\{\begin{array}{cc} \alpha_{xK}\left(1\right) & \mathrm{if}W_{x}\leq W_{xfsr(k-1)}\\ \alpha_{xK}\left(W_{x}\right) & \mathrm{if}W_{xfsr(k-1)}<W_{x}\leq W_{xfsr(k)}\\ 1 & \mathrm{if}W_{x}>W_{xfsr(k)} \end{array}\right.$$  12. Save all membership functions and label them as: $MFW_{j}=mfw_{x1}$,…, $MFW_{j+K-1}$ = $mfw_{xK}$, and $MFT_{j}=mft_{x1}$,…, $MFT_{j+K-1}=mft_{xK}$.  13. If the wavelength positions of another fringe peak will be considered as an input, then increment the *j* counter to j=j+K and repeat steps 2 to 12.


As an example of the application of Algorithm 3, the membership functions for the case of the synthetic dataset of sensor 1 will be built. Here, the wavelength positions $W_{2}$ of the fringe peak $P_{2}$ will be considered as the input of FIS2. Moreover, as was discussed previously, there occur two FSR periods for the temperature range 0 ≤ *T* ≤ 70 °C. The first FSR period will occur when the temperature is within the range from $T_{fsr(0)}$ = 0 and $T_{fsr(1)}$ = 40 °C. The second FSR period will be within $T_{fsr(1)}$ = 40 and $T_{fsr(2)}$ = 70 °C (Figure 6a). Therefore, for this case, *K* = 2 and consequently, all observations are divided into groups $OG_{1}$ and $OG_{2}$ (Figure 6a). Later, observations of each group were fitted to obtain datasets [$T_{21}$, $W_{21}$] and [$T_{22}$, $W_{22}$]. Afterwards, the column arrays $T_{xK}$ of the dataset were normalized between (0,1] to form the column $\alpha_{xK}$ and the *K* dataset [$T_{21}$, $W_{21}$, $\alpha_{21}$] and [$T_{22}$, $W_{22}$, $\alpha_{22}$]. Now, the minimum and maximum wavelength positions of fringe $P_{2}$ were localized between the $W_{2K}$ columns of the datasets to set the universe of discourse for this input as $U_{W_{2}}$ = [$W_{2\min}-\Delta W_{2}$, $W_{2\max}+\Delta W_{2}$] = [1504.24,1508.85], here $\Delta W_{2}$ = 0.1 nm. Taking as a reference this universe of discourse and by using Equation (7), the functions $mfw_{21}$ and $mfw_{22}$ were formed (Figure 6b). Later, membership functions $mft_{21}$ and $mft_{22}$ were built as described by Equation (6) (Figure 6c). Finally, these functions were saved as $MFT_{1}=mft_{21}$ and $MFT_{2}=mft_{22}$, and the final counter’s value was set to j=2.

#### Inference Mechanism Used in FIS2

The implication in FIS2 was evaluated with the Tsukamoto inference mechanism [26]. Here, the set of membership functions $MFW_{j}$ formed by means of Algorithm 3 were used to fuzzificate the wavelength positions of fringes that were considered as inputs. Additionally, in order to fuzzificate the other input of FIS2, the period of FSR ($pfsr_{o}$), the set of membership functions $MFO_{1}$ formed with Algorithm 2 and described in the FIS1 section was used. Moreover, the $MFT_{j}$ membership functions, formed with Algorithm 3 will be used to evaluate the consequences of the rules. Afterwards, as usual, a set of *j* IF–THEN fuzzy rules must be defined. Later, each one of these rules can be evaluated numerically by firstly determining its corresponding firing levels [26], which can be obtained by:

(8) $$\alpha_{j}=\min\left[MFO_{1}^{\tau }\left(fsr_{o}\right),MFW_{j}\left(W_{xo}\right)\right]$$

where τ=mod((j−1)/*k*)+1. Moreover, the relationship between the *j*-th estimated temperature and the firing level $\alpha_{j}$ for each fuzzy rule is given by:

(9) $$MFT_{j}\left(T_{\mathrm{est}j}\right)=\alpha_{j}$$

Finally, according to the Tsukamoto inference mechanism, all the rules’ consequences are aggregated, and its corresponding discrete center-of-gravity can be computed by using Equation (10) [26]:

(10) $$T_{\mathrm{est}}\left(W_{xo},fsr_{o}\right)=\frac{\sum_{h=1}^{j}T_{\mathrm{est}h}\alpha_{h}}{\sum_{h=1}^{j}\alpha_{h}}$$

**Figure 7:**
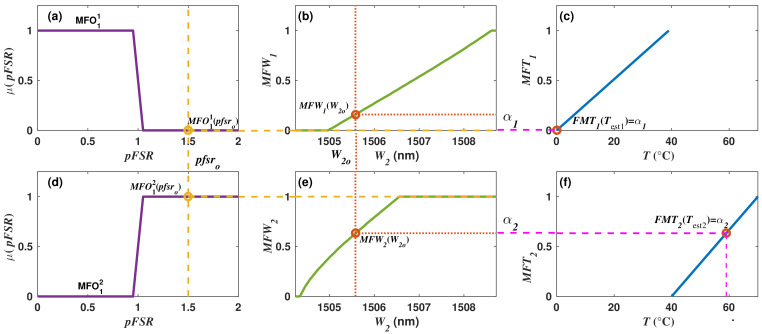
Example of the membership functions built with Algorithm 3 for the case when $W_{2}$ is considered as the input and the inference mechanisms procedure. (**a**,**d**) membership functions used to fuzzificate the input variable pFSRo; (**b**,**e**) membership functions used to fuzzificate the input variable $W_{2}$, for the particular value of ($W_{2o}$); at the right shows the corresponding firing levels for this case ($\alpha_{1}$ and $\alpha_{2}$); (**c**,**f**) membership functions $MFT_{1}$ and $MFT_{2}$ used to evaluate the consequences of the two fuzzy rules.

As an example of the application of this inference mechanism, let us continue using the data of the particular observation of the synthetic dataset of filter 1 and given in Table 2. For this observation, the wavelength positions of the peak *x* = 2 is $W_{2o}$ = 1505.58 nm, and it will be one of the two inputs of FIS2. This input will be fuzzificated with the membership functions $MFW_{1}$ and $MFW_{2}$, and it will be obtained that $MFW_{1}\left(1505.58\right)$ = 0.159 (Figure 7b) and $MFW_{2}\left(1505.58\right)$ = 0.633 (Figure 7e). Moreover, the second input variable of FIS2 is the pFSRo obtained in FIS1; for this particular observation, it was obtained previously that pFSRo = 1.5. This input is fuzzificated with the membership functions $MFO_{1}^{1}$(pFSRo) = $MFO_{1}^{1}$(1.5) = 0 (Figure 7a) and $MFO_{1}^{2}$(pFSRo) = $MFO_{1}^{2}$(1.5) = 1 (Figure 7d). Afterwards, the firing levels and the consequences for each fuzzy rule (Table 3) can be calculated. Here, firing levels are computed by using Equation (8), and it is obtained for the rule 1 that $\alpha_{1}$ = min($MFW_{1}\left(1505.58\right)$, $MFO_{1}^{1}$(1.5)) = min(0.159,0) = 0 (Figure 7b). Similarly, the firing level corresponding the second fuzzy rule is $\alpha_{2}$ = min($MFW_{2}\left(1505.58\right)$, $MFO_{1}^{2}$(1.5)) = min(0.633,1) = 0.633 (Figure 7d). Later, the output of each fuzzy rule is calculated by using Equation (9). For the first fuzzy rule, we obtain $MFT_{1}\left(T_{\mathrm{est}1}\right)=MFT_{1}$(0 °C) = $\alpha_{1}$ = 0 (Figure 7c), and for the second fuzzy rule, $MFT_{2}\left(T_{\mathrm{est}2}\right)=FMT_{2}$(59.005 °C) = $\alpha_{2}$ = 0.633 (Figure 7f). All fuzzy rules outputs are aggregated and deffuzified by using Equation (10), and for this particular example, it is obtained that:

(11) $$T_{\mathrm{est}}\left(1505.58,1.5\right)=\frac{T_{\mathrm{est}1}\alpha_{1}+T_{\mathrm{est}2}\alpha_{2}}{\alpha_{1}+\alpha_{2}}=\frac{\left(0\times 0)+(59.005\times 0.633\right)}{0+0.633}=59.005{\;}^{{}^{\circ}}C$$

For the observation considered in this example, the reference temperature was 59 °C (Table 3), and therefore, the absolute error (AE) of the estimated with the proposed FIS was 0.005 °C. The result for this example shows the viability of the proposed methodology to estimate the temperature over more than one period of FSR, overcoming the 2π ambiguity.

## 4. Results

This section presents the results obtained when the proposed methodology was applied to synthetic and experimental datasets of two different filters. Both synthetic datasets were formed by extracting features of simulated filter spectra, which were computed by using the mathematical model proposed previously by [25]. Experimental datasets were formed with data extracted from measured spectra [25].

### 4.1. Filter 1: Synthetic Dataset

**Figure 8:**
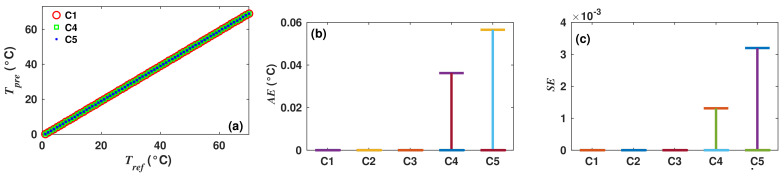
(**a**) Reference and estimated temperatures obtained with the methodology for the synthetic dataset of filter 1; (**b**,**c**) corresponding AE and SE distributions obtained with different inputs combinations.

This dataset contains 71 observations, covering the temperature range from 0 to 70 °C, with steps of 1 °C. Moreover, in each observation, the wavelength positions of six peaks ($W_{1}$ to $W_{6}$) were registered, as were the ratio of amplitudes of peaks 1 and 2 ($r_{12}$) and finally the reference temperature ($T_{ref}$). An example of one row (observation) of the dataset is shown in Table 2. Now, according to the methodology, FIS1 must provide the number of FSR periods (pFSRo) corresponding to the value of the input $r_{12}$. FIS2 is the one that determines the overall estimated temperature ($T_{\mathrm{est}}$) based on the combination of inputs values. In the proposed methodology, the inputs of FIS2 are pFSRo and one or more wavelength positions of different peaks. In our case, the $T_{\mathrm{est}}$ was determined considering different combinations of wavelength positions as inputs. For each combination, the mean absolute error (MAE) and mean squared error (MSE) between the $T_{ref}$ and $T_{\mathrm{est}}$ were evaluated. Here, it was considered that those combinations presenting the lower MSE are the better choices to estimate the temperature. Table 4 lists the five combinations of inputs that better estimated the temperature. For this dataset, input combinations C1, C2 and C3 produced quasi perfect fits (Figure 8a) with MAE and MSE values of practically zero. Moreover, with inputs combinations C4, C5 and C6, the MAE is <0.0035 °C, which is considerably low, and the corresponding AE and SE distributions for the five inputs combinations are shown in Figure 8b,c, respectively. From these numerical results, it can be observed that all observations were correctly processed by our proposed methodology, and it allowed us to estimate correctly the temperature over two FSR periods.

### 4.2. Filter 1: Experimental Dataset

**Figure 9:**
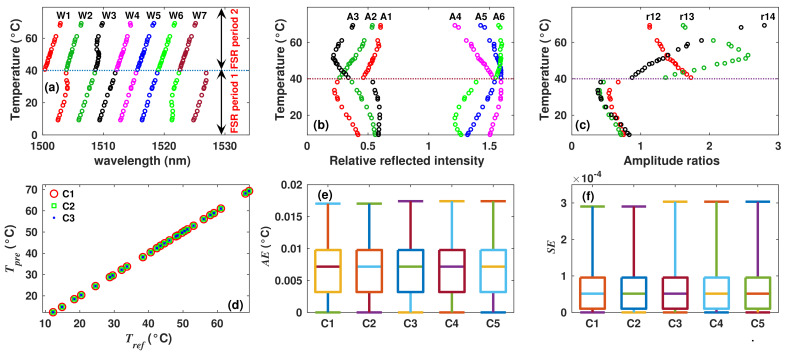
Measured (**a**) peak wavelength positions, (**b**) peak amplitudes, (**c**) amplitudes ratio $r_{12}$ as a function of temperature of sensor 1. In (**b**), A5–A7 were shifted 1 unit to the right for clarity purposes; (**d**) comparison between $T_{\mathrm{est}}$ and $T_{ref}$; (**e**) AE and (**f**) SE distributions for $T_{\mathrm{est}}$ obtained considering different input combinations cases.

In this second case, the proposed methodology was applied to a dataset formed with experimental data. In general terms, the filter spectrum can be well described by the mathematical model used to form the synthetic dataset analyzed in the previous section. However, experimental spectra show slight differences in amplitudes (Figure 9b) and wavelength positions of the fringe peaks (Figure 9a). In this dataset, only 29 observations were registered for temperatures within 9 and 70 °C. The proposed methodology is robust to these small variations, since it does not depend directly on precise values of the peak amplitudes; here, the general behavior of the amplitude ratio $r_{12}$ is more important. From Figure 9c, it can be observed that $r_{12}$ < 1 within the first pSFR period (*T* ≤ 40 ${}^{{}^{\circ}}$C). In contrast, $r_{12}$ > 1 within the second pSFR period (40 < *T* ≤ 70 ${}^{{}^{\circ}}$C). These are the same characteristics observed in the simulated case, and therefore, it is possible to use the same fuzzy rules and the membership functions formed for the FIS1 of the synthetic dataset of this filter. Moreover, Algorithm 3 must be performed in order to fit the wavelength positions and temperatures for each one of the FSR periods and obtain the membership functions $MFW_{1}$, $MFW_{2}$, $MFT_{1}$ and $MFT_{2}$. Later, the fuzzy rules described in Table 3 were evaluated by using Equations (8) and (9). Finally, the estimated temperatures were determined by means of Equation (10) and by considering combinations of wavelength position of different peaks. For this experimental sensor, the best estimation was obtained when the variables $W_{2}$, $W_{6}$ and $r_{12}$ (case C1) were considered as inputs of the system. For case C1, the MAE = 7.4 × 10${}^{-3}$ ${}^{{}^{\circ}}$C, while the MSE = 8.1 × 10${}^{-5}$ [${}^{{}^{\circ}}$C]${}^{2}$. Furthermore, the MSE and MAE for the other four inputs combinations are listed in Table 4, while their corresponding AE and SE errors are presented in Figure 9b,c, respectively. Finally, these results show that the proposed methodology is able to determine correctly and with relatively high precision the temperature over two FSR periods. This means that it is possible to widen the typical measurement range of similar interferometric sensors.

### 4.3. Filter 2: Synthetic Dataset

**Figure 10:**
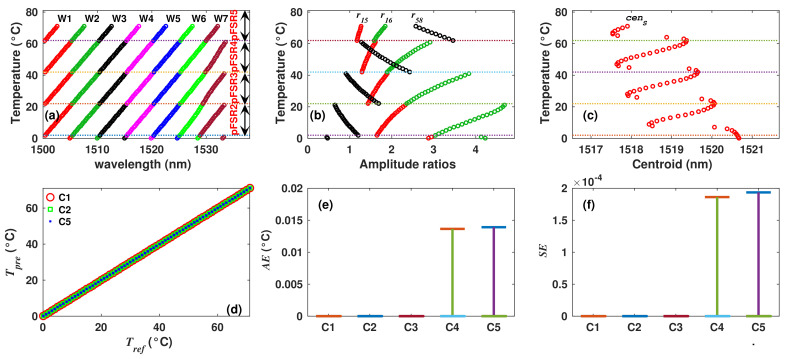
Simulated (**a**) peaks wavelength positions, (**b**) some amplitude ratios and (**c**) centroid of the spectrum as a function of temperature of sensor 2; (**d**) comparison between $T_{\mathrm{est}}$ and $T_{ref}$; (**e**) AE and (**f**) SE distributions of the for $T_{\mathrm{est}}$ obtained considering different input combinations.

The dataset for this case was formed by simulating the spectra of filter 2 for temperatures between 0 ≤ *T* ≤ 70 ${}^{{}^{\circ}}$C with steps of 1 ${}^{{}^{\circ}}$C. From simulated spectra, we extracted the features: wavelength positions of 10 peaks ($W_{1}$ to $W_{10}$), the amplitude ratios $r_{15}$, $r_{16}$, $r_{58}$ and the centroid of the spectrum ($cen_{S}$). Moreover, when the temperature is plotted as a function of the wavelength peak positions, it can be clearly observed that five FSR periods occur within this temperature range (Figure 10a). The first FSR period occurs between 0 ≤ *T* < 2 ${}^{{}^{\circ}}$C, the second between 2 ≤ *T* < 22 ${}^{{}^{\circ}}$C, the third between 22 ≤ *T* < 42 ${}^{{}^{\circ}}$C, the fourth between 42 ≤ *T* < 62 ${}^{{}^{\circ}}$C and the fifth between 62 ≤ *T* ≤ 70 ${}^{{}^{\circ}}$C. Therefore, this sensor is more complex than sensor 1 analyzed previously, since now, it is required to determine correctly the temperature over five FSR periods. Here, the methodology described previously will be applied exactly in the same way as for filter 1. The main difference lies in the number of inputs needed to estimate the temperature. In our case, $r_{15}$, $r_{16}$, $r_{58}$ (Figure 10b) and $cen_{S}$ (Figure 10c) were selected as inputs of FIS1. The membership functions used to fuzzificate these inputs were built following Algorithm 1 of the methodology. The universes of discourse of variables $r_{15}$, $r_{16}$ and $r_{58}$ were partitioned into 10 regions, while the universe of discourse of variable $cen_{S}$ was partitioned into five regions. Moreover, the universe of discourse of the output variable of FIS1 was set as $U_{pFSR}=\left[0,5\right]$, and it was partitioned to form five membership functions following Algorithm 2. In order to apply the inference mechanism of FIS1, a set of 19 fuzzy rules were defined and are listed in Table 5. These rules allowed us to determine correctly the period of FSR (pFSRo) for each observation of the dataset. Later, pFSRo is used as an input of FIS2 in combination with one or more peak’s wavelength positions. After, membership functions required to implement FIS2 were built following Algorithm 3, and finally, the temperature was estimated by using the Tsukamoto inference mechanism described previously. For the synthetic dataset of sensor 2, the best temperature estimations were obtained when variables combinations C1, C2 and C3 were considered. For these cases, the corresponding MAE and MSE were practically zero (Table 6), showing a quasi perfect fit between $T_{\mathrm{est}}$ and $T_{ref}$ (Figure 10d). Moreover, Figure 10e,f show the AE and SE distributions obtained for five different input combinations. These results are important because they demonstrate that by applying this methodology, the measurement range can be increased by a factor of 5, since it can determine the temperature over at least five FSR periods.

### 4.4. Filter 2: Experimental Dataset

**Figure 11:**
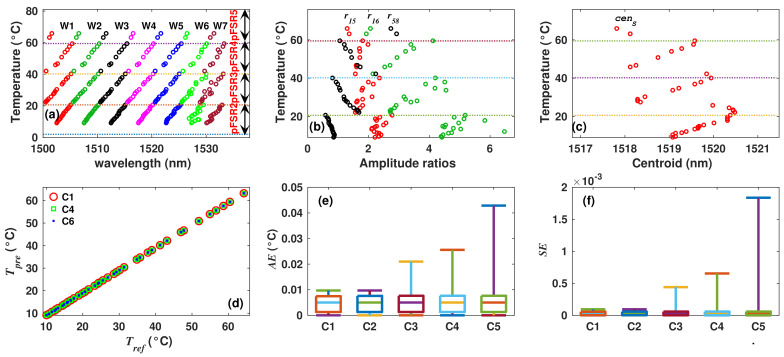
Experimentally measured (**a**) peaks wavelength positions, (**b**) some amplitude ratios and (**c**) centroid of the spectrum as a function of temperature of sensor 2; (**d**) comparison between $T_{\mathrm{est}}$ and $T_{ref}$; (**e**) AE and (**f**) SE distributions of the $T_{\mathrm{est}}$ obtained considering different input combinations.

For this case, the experimental sensing arrangement was implemented with the filter 2, and there were recorded 40 spectra between the temperature range from 9 ≤ *T* ≤ 70 °C. From these spectra, we extracted the $W_{1}$ to $W_{10}$ (Figure 11a), $r_{15}$, $r_{16}$, $r_{58}$ (Figure 11b) and $cen_{S}$ (Figure 11c) features to form the observations of the dataset. Here, when the wavelength positions of fringe peaks are traced as a function of the temperature, it can be observed that these behave in general terms, as it was described by the simulated results presented in the previous section. Moreover, for the temperature range used in our experiments, there can be observed four FSR periods (Figure 11a) that match with four of the periods described for the synthetic case, these are: the second between 2 ≤ *T* < 22 °C, the third between 22 ≤ *T* < 42 °C, the fourth between 42 ≤ *T* < 62 °C and the fifth between 62 ≤ *T* ≤ 70 °C. Therefore, the system was evaluated in a similar way as in the case of the synthetic dataset of this sensor. Here, to fuzzificate the inputs variables $r_{15}$, $r_{16}$ and $r_{58}$, their corresponding universes of discourse were partitioned into 26 regions. The universe of discourse of the variable $cen_{S}$ was partitioned into five regions. Afterwards, their corresponding membership functions there were formed by performing Algorithm 1. Moreover, the universe of discourse of the output variable of FIS1 was partitioned in five regions (one for each FSR period), and its corresponding membership functions there were built executing Algorithm 2. The period of FSR ($pFSR_{o}$) was determined by evaluating the fuzzy rules listed in Table 5 and by applying Mamdani’s inference mechanism. Afterwards, the membership functions required to implement FIS2 there were formed by means of Algorithm 3. Finally, the temperature was estimated considering different combinations of inputs of FIS2. Here, the best case (C1) was obtained when $W_{3}$, $W_{4}$, $W_{9}$, $r_{15}$, $r_{16}$, $r_{58}$ and $cen_{S}$ there were selected as inputs. For case C1, the MAE = 0.0045 °C and MSE = 3 × 10${}^{-5}$[°C]${}^{2}$. Moreover, other inputs combinations produced similar MAE and MSE values (Table 6), and the quality of the fit can be appreciated in Figure 11c. Moreover, AE and SE distributions for five different inputs combinations are shown in Figure 11e,f, respectively.

## 5. Discussion

In this work, we presented a methodology, based on a fuzzy inference system, which can make it possible to perform measurements with an interferometric sensor abroad the typical limit of 1 free spectral range. The relevance of this methodology is that the typical 2π ambiguity of interferometric sensors can be overcome, which is not possible when the typical way of establishing a relationship between the measurand and wavelength position of one fringe’s peak or dip is used. In order to demonstrate the viability of the methodology, two temperature sensing arrangements were implemented. For implementing each sensor, different interferometric filters were used. The spectra of these filters were both simulated and experimentally measured at different temperatures. From these spectra, some features were extracted to form a synthetic and experimental datasets for each sensing arrangement. Furthermore, it was demonstrated that by using the methodology presented in this work, it was possible to determine temperature over five FSR periods for sensor 2, which implies that its measurement range was increased by a factor of 5. For sensor 1, the temperature was successfully estimated over 2 FSR periods, meaning that its corresponding measurement range was doubled. These results also support the fact that the measurement ambiguity can be overcome within a large number of FSR periods by only considering more spectral features as input variables of the FIS. Finally, it was shown that the estimation of the temperature is relatively high, since for sensor 1, the temperature within 2 FSR periods was determined correctly with an MSE = 8.1 × 10${}^{-5}$[${}^{{}^{\circ}}$C]${}^{2}$. For sensor 2, the measurement range was quintupled: the MSE = 3 × 10${}^{-5}$[${}^{{}^{\circ}}$C]${}^{2}$. Finally, for future work, we will analyzed the potential of this methodology to enhance the sensor sensitivity and to sensing different parameters simultaneously such as temperature and refractive index.

## Figures and Tables

**Table 1:** Example of fuzzy rules of FIS1. These were defined for sensor 1.

Number of Rule		Rule Description		
1	IF $r_{12o}$ is ($MFI_{1}^{1}$ or $MFI_{1}^{2}$ or $MFI_{1}^{3}$ or $MFI_{1}^{4}$ or $MFI_{1}^{5}$) THEN $MFO_{1}^{1}$			
2	IF $r_{12o}$ is ($MFI_{1}^{6}$ or $MFI_{1}^{7}$ or $MFI_{1}^{8}$ or $MFI_{1}^{9}$ or $MFI_{1}^{10}$) THEN $MFO_{1}^{2}$			

**Table 2:** Example of an observation of the synthetic dataset of filter 1.

$W_{1}$ (nm)	$W_{2}$ (nm)	$W_{3}$ (nm)	$W_{4}$ (nm)	$W_{5}$ (nm)	$W_{6}$ (nm)	$r_{12}$	$T_{\mathrm{ref}}$
1502.13	1505.58	1509.73	1513.97	1517.50	1520.91	1.379	59

**Table 3:** Definition of the fuzzy rules of FIS2.

Number of Rule		Rule Description		
1	IF [($W_{1o}$ is $MFW_{1}$) AND ($fsr_{o}$ is $MFO_{1}^{1}$)] THEN $MFT_{1}$			
⋮	⋮			
*j*	IF [($W_{1o}$ is $MFW_{j}$) AND ($fsr_{o}$ is $MFO_{1}^{\tau }$)] THEN $MFT_{j}$			

**Table 4:** MAE and MSE obtained for different combinations of inputs in the FIS for synthetic and experimental datasets of sensor 1.

	Synthetic Dataset			Experimental Dataset		
Case Label	Inputs	MAE (${}^{{}^{\circ}}$C)	MSE ((${}^{{}^{\circ}}$C)${}^{2}$)	Inputs	MAE (${}^{{}^{\circ}}$C)	MSE ((${}^{{}^{\circ}}$C)${}^{2}$)
C1	$W_{4}$, $r_{12}$	4.2 × 10${}^{-14}$	1.2 × 10${}^{-25}$	$W_{2}$, $W_{6}$, $r_{12}$	7.4 × 10${}^{-3}$	8.1 × 10${}^{-5}$
C2	$W_{6}$, $r_{12}$	4.2 × 10${}^{-14}$	1.2 × 10${}^{-25}$	$W_{5}$, $W_{6}$, $r_{12}$	7.4 × 10${}^{-3}$	8.1 × 10${}^{-5}$
C3	$W_{4}$, $W_{6}$, $r_{12}$	4.2 × 10${}^{-14}$	1.3 × 10${}^{-25}$	$W_{2}$, $r_{12}$	7.5 × 10${}^{-3}$	8.4 × 10${}^{-5}$
C4	$W_{3}$, $W_{4}$, $W_{6}$, $r_{12}$	3.4 × 10${}^{-3}$	1.2 × 10${}^{-4}$	$W_{5}$, $r_{12}$	7.5 × 10${}^{-3}$	8.4 × 10${}^{-5}$
C5	$W_{1}$, $W_{4}$, $r_{12}$	2.2 × 10${}^{-3}$	1.2 × 10${}^{-4}$	$W_{2}$, $W_{5}$, $r_{12}$	7.5 × 10${}^{-3}$	8.4 × 10${}^{-5}$

**Table 5:** Inference Rules of the FIS1 for Sensor 2.

	Synthetic Dataset					Experimental Dataset				
Number of Rule	$r_{15o}$	$r_{16o}$	$r_{58o}$	${\mathrm{cen}}_{\mathrm{So}}$	pFSR	$r_{15o}$	$r_{16o}$	$r_{58o}$	${\mathrm{cen}}_{\mathrm{So}}$	pFSR
R1	$MFI_{1}^{6}$	$MFI_{2}^{9}$	$MFI_{3}^{1}$	$MFI_{4}^{5}$	$MFO_{1}^{1}$	$MFI_{1}^{9}$	$MFI_{2}^{18}$	$MFI_{3}^{4}$	$MFI_{4}^{3}$	$MFO_{1}^{2}$
R2	$MFI_{1}^{4}$	$MFI_{2}^{7}$	$MFI_{3}^{3}$	$MFI_{4}^{5}$	$MFO_{1}^{2}$	$MFI_{1}^{9}$	$MFI_{2}^{19\;\mathrm{OR}\;20}$	$MFI_{3}^{4}$	$MFI_{4}^{4}$	$MFO_{1}^{2}$
R3	$MFI_{1}^{4}$	$MFI_{2}^{8}$	$MFI_{3}^{2}$	$MFI_{4}^{2}$	$MFO_{1}^{2}$	$MFI_{1}^{9}$	$MFI_{2}^{21}$	$MFI_{3}^{3}$	$MFI_{4}^{5}$	$MFO_{1}^{2}$
R4	$MFI_{1}^{4}$	$MFI_{2}^{9}$	$MFI_{3}^{2}$	$MFI_{4}^{3}$	$MFO_{1}^{2}$	$MFI_{1}^{9}$	$MFI_{2}^{21}$	$MFI_{3}^{4}$	$MFI_{4}^{4}$	$MFO_{1}^{2}$
R5	$MFI_{1}^{5}$	$MFI_{2}^{9}$	$MFI_{3}^{2}$	$MFI_{4}^{3\;\mathrm{OR}\;4}$	$MFO_{1}^{2}$	$MFI_{1}^{9}$	$MFI_{2}^{26}$	$MFI_{3}^{4}$	$MFI_{4}^{3}$	$MFO_{1}^{2}$
R6	$MFI_{1}^{5}$	$MFI_{2}^{10}$	$MFI_{3}^{2}$	$MFI_{4}^{4}$	$MFO_{1}^{2}$	$MFI_{1}^{10}$	$MFI_{2}^{18}$	$MFI_{3}^{4}$	$MFI_{4}^{3\;\mathrm{OR}\;4}$	$MFO_{1}^{2}$
R7	$MFI_{1}^{3}$	$MFI_{2}^{5}$	$MFI_{3}^{4}$	$MFI_{4}^{4\;\mathrm{OR}\;5}$	$MFO_{1}^{3}$	$MFI_{1}^{10}$	$MFI_{2}^{19}$	$MFI_{3}^{3}$	$MFI_{4}^{4}$	$MFO_{1}^{2}$
R8	$MFI_{1}^{3}$	$MFI_{2}^{6}$	$MFI_{3}^{4}$	$MFI_{4}^{4}$	$MFO_{1}^{3}$	$MFI_{1}^{10}$	$MFI_{2}^{19}$	$MFI_{3}^{4}$	$MFI_{4}^{3}$	$MFO_{1}^{2}$
R9	$MFI_{1}^{4}$	$MFI_{2}^{6}$	$MFI_{3}^{3}$	$MFI_{4}^{1\;\mathrm{OR}\;2}$	$MFO_{1}^{3}$	$MFI_{1}^{10}$	$MFI_{2}^{20\;\mathrm{OR}\;24}$	$MFI_{3}^{3}$	$MFI_{4}^{5}$	$MFO_{1}^{2}$
R10	$MFI_{1}^{4}$	$MFI_{2}^{7}$	$MFI_{3}^{3}$	$MFI_{4}^{2\;\mathrm{OR}\;3}$	$MFO_{1}^{3}$	$MFI_{1}^{11}$	$MFI_{2}^{20}$	$MFI_{3}^{3}$	$MFI_{4}^{5}$	$MFO_{1}^{2}$
R11	$MFI_{1}^{4}$	$MFI_{2}^{8}$	$MFI_{3}^{2}$	$MFI_{4}^{3\;\mathrm{OR}\;4}$	$MFO_{1}^{3}$	$MFI_{1}^{12}$	$MFI_{2}^{21}$	$MFI_{3}^{3}$	$MFI_{4}^{5}$	$MFO_{1}^{2}$
R12	$MFI_{1}^{3}$	$MFI_{2}^{4}$	$MFI_{3}^{5}$	$MFI_{4}^{4}$	$MFO_{1}^{4}$	$MFI_{1}^{7}$	$MFI_{2}^{11}$	$MFI_{3}^{7}$	$MFI_{4}^{5}$	$MFO_{1}^{3}$
R13	$MFI_{1}^{3}$	$MFI_{2}^{5}$	$MFI_{3}^{4}$	$MFI_{4}^{1\;\mathrm{OR}\;2}$	$MFO_{1}^{4}$	$MFI_{1}^{7}$	$MFI_{2}^{12}$	$MFI_{3}^{7}$	$MFI_{4}^{5}$	$MFO_{1}^{3}$
R14	$MFI_{1}^{3}$	$MFI_{2}^{5}$	$MFI_{3}^{5}$	$MFI_{4}^{1}$	$MFO_{1}^{4}$	$MFI_{1}^{7}$	$MFI_{2}^{14}$	$MFI_{3}^{6}$	$MFI_{4}^{2}$	$MFO_{1}^{3}$
R15	$MFI_{1}^{3}$	$MFI_{2}^{6}$	$MFI_{3}^{4}$	$MFI_{4}^{2}$	$MFO_{1}^{4}$	$MFI_{1}^{7}$	$MFI_{2}^{15}$	$MFI_{3}^{6}$	$MFI_{4}^{1}$	$MFO_{1}^{3}$
R16	$MFI_{1}^{4}$	$MFI_{2}^{6}$	$MFI_{3}^{3}$	$MFI_{4}^{3}$	$MFO_{1}^{4}$	$MFI_{1}^{8}$	$MFI_{2}^{12}$	$MFI_{3}^{6}$	$MFI_{4}^{5}$	$MFO_{1}^{3}$
R17	$MFI_{1}^{4}$	$MFI_{2}^{6}$	$MFI_{3}^{6}$	$MFI_{4}^{2}$	$MFO_{1}^{4}$	$MFI_{1}^{8}$	$MFI_{2}^{13}$	$MFI_{3}^{5}$	$MFI_{4}^{1}$	$MFO_{1}^{3}$
R18	$MFI_{1}^{3}$	$MFI_{2}^{4}$	$MFI_{3}^{6}$	$MFI_{4}^{1}$	$MFO_{1}^{5}$	$MFI_{1}^{8}$	$MFI_{2}^{19}$	$MFI_{3}^{4}$	$MFI_{4}^{4}$	$MFO_{1}^{3}$
R19	$MFI_{1}^{3}$	$MFI_{2}^{4}$	$MFI_{3}^{7}$	$MFI_{4}^{1\;\mathrm{OR}\;3}$	$MFO_{1}^{5}$	$MFI_{1}^{8}$	$MFI_{2}^{17\;\mathrm{OR}\;19}$	$MFI_{3}^{5}$	$MFI_{4}^{3}$	$MFO_{1}^{3}$
R20						$MFI_{1}^{9}$	$MFI_{2}^{13}$	$MFI_{3}^{5}$	$MFI_{4}^{2}$	$MFO_{1}^{3}$
R21						$MFI_{1}^{9}$	$MFI_{2}^{16}$	$MFI_{3}^{4}$	$MFI_{4}^{4}$	$MFO_{1}^{3}$
R22						$MFI_{1}^{10}$	$MFI_{2}^{17}$	$MFI_{3}^{4}$	$MFI_{4}^{5}$	$MFO_{1}^{3}$
R23						$MFI_{1}^{7}$	$MFI_{2}^{9}$	$MFI_{3}^{9}$	$MFI_{4}^{4}$	$MFO_{1}^{4}$
R24						$MFI_{1}^{7}$	$MFI_{2}^{11}$	$MFI_{3}^{7}$	$MFI_{4}^{1}$	$MFO_{1}^{4}$
R25						$MFI_{1}^{7}$	$MFI_{2}^{12}$	$MFI_{3}^{6}$	$MFI_{4}^{2}$	$MFO_{1}^{4}$
R26						$MFI_{1}^{7}$	$MFI_{2}^{13}$	$MFI_{3}^{6}$	$MFI_{4}^{3}$	$MFO_{1}^{4}$
R27						$MFI_{1}^{7}$	$MFI_{2}^{14}$	$MFI_{3}^{5}$	$MFI_{4}^{3}$	$MFO_{1}^{4}$
R28						$MFI_{1}^{8}$	$MFI_{2}^{14\;\mathrm{OR}\;17}$	$MFI_{3}^{5}$	$MFI_{4}^{4}$	$MFO_{1}^{4}$
R29						$MFI_{1}^{6}$	$MFI_{2}^{8}$	$MFI_{3}^{12}$	$MFI_{4}^{1}$	$MFO_{1}^{5}$
R30						$MFI_{1}^{6}$	$MFI_{2}^{9}$	$MFI_{3}^{11}$	$MFI_{4}^{1}$	$MFO_{1}^{5}$

**Table 6:** MAE and MSE obtained for different combinations of inputs in the FIS for synthetic and experimental datasets of sensor 2.

	Synthetic Dataset			Experimental Dataset		
Case Label	Inputs	MAE (°C)	MSE ((°C)${}^{2}$)	Inputs	MAE (°C)	MSE ((°C)${}^{2}$)
	$(r_{15}$, $r_{16}$, $r_{58}$, ${\mathrm{cen}}_{S})$			$(r_{15}$, $r_{16}$, $r_{58}$, ${\mathrm{cen}}_{S})$		
C1	$W_{9}$	0	0	$W_{3}$,$W_{4}$,$W_{9}$	4.5 × 10${}^{-3}$	3.0 × 10${}^{-5}$
C2	$W_{10}$	0	0	$W_{2}$, $W_{4}$, $W_{9}$	4.5 × 10${}^{-3}$	3.1 × 10${}^{-5}$
C3	$W_{9}$, $W_{10}$	0	0	$W_{3}$, $W_{9}$	4.8 × 10${}^{-3}$	4.0 × 10${}^{-5}$
C4	$W_{4}$, $W_{9}$, $W_{10}$	3.7 × 10${}^{-4}$	5.0 × 10${}^{-6}$	$W_{4}$, $W_{9}$, $W_{10}$	5.0 × 10${}^{-3}$	4.6 × 10${}^{-5}$
C5	$W_{5}$, $W_{9}$, $W_{10}$	3.8 × 10${}^{-4}$	5.3 × 10${}^{-6}$	$W_{2}$, $W_{9}$	5.4 × 10${}^{-3}$	7.5 × 10${}^{-5}$
